# Experimental Drought Suppresses Amphibian Pathogen Yet Intensifies Transmission and Disrupts Protective Skin Microbiome

**DOI:** 10.1111/gcb.70275

**Published:** 2025-06-06

**Authors:** Shannon Buttimer, Daniel Medina, Renato A. Martins, Ana Gabrielle Morais da Silva, Wesley J. Neely, Célio F. B. Haddad, Graziella V. DiRenzo, Alessandro Catenazzi, Rayna C. Bell, C. Guilherme Becker

**Affiliations:** ^1^ Department of Biology The Pennsylvania State University University Park Pennsylvania USA; ^2^ One Health Microbiome Center Center for Infectious Disease Dynamics, Huck Institutes of the Life Sciences The Pennsylvania State University University Park Pennsylvania USA; ^3^ Department of Biodiversity, Aquaculture Center (CAUNESP), and CBioClima, I.B. Universidade Estadual Paulista Rio Claro São Paulo Brazil; ^4^ Centro Universitário Nossa Senhora do Patrocínio (CEUNSP) Itú São Paulo Brazil; ^5^ Department of Biology Texas State University San Marcos Texas USA; ^6^ U.S. Geological Survey, Massachusetts Cooperative Fish and Wildlife Research Unit University of Massachusetts Amherst Massachusetts USA; ^7^ Department of Biological Sciences Florida International University Miami Florida USA; ^8^ Department of Herpetology California Academy of Sciences San Francisco California USA

**Keywords:** amphibian, *Batrachochytrium dendrobatidis*, *Brachycephalus pitanga*, climate change, disease, drought, microbiome, tropical ecology

## Abstract

Shifting precipitation regimes driven by global climate change can alter vertebrate behavior and host‐symbiont relationships, potentially compromising host resistance to pathogen invasion. In Brazil's Atlantic Forest, a biodiversity hotspot, prior research identified drought as a key factor disrupting the skin microbiome, contributing to a die‐off of pumpkin toadlets due to the invasive waterborne fungal pathogen *Batrachochytrium dendrobatidis* (Bd). However, observational studies cannot disentangle the direct effect of moisture on Bd growth from increased amphibian activity during wet breeding seasons. Using field enclosures, we experimentally tested the influence of drought conditions on host microhabitat use, Bd disease dynamics, and the composition and predicted Bd‐inhibitory function of cutaneous bacterial communities. Each enclosure housed ecologically realistic densities of *Brachycephalus pitanga* , a micro‐endemic pumpkin toadlet. We simulated a short‐term drought in half of the enclosures using translucent tarp coverings. To track individual toadlets, we identified their unique markings and collected skin swabs biweekly over 3 months. We then implemented molecular techniques to quantify Bd loads and characterize skin bacterial diversity and composition over time. Our findings indicate that while drought may reduce overall Bd loads on hosts, this effect is partially offset by an increase in the use of water‐filled areas of the enclosures and by a disruption of the protective host skin microbiome. This study provides valuable insights into the cascading impacts of climate change on animal behavior, host‐symbiont interactions, and disease dynamics.

## Graphical abstract

This study used ecologically realistic enclosures with pumpkin toadlets to simulate short‐term drought conditions in Brazil's Atlantic Forest. Drought reduced the overall levels of a harmful amphibian fungus, but it also disrupted their protective skin‐associated bacteria and caused the toadlets to cluster in wet areas of the enclosures, which increased their risk of infection by the waterborne fungus. These results highlight the complex ways that drought can influence disease risk in wildlife.

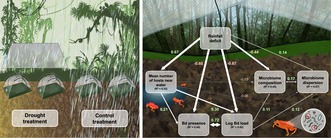

## BRAZILIAN PORTUGUESE ABSTRACT

Mudanças nos regimes de chuva causadas pelas mudanças climáticas podem alterar o comportamento de vertebrados e interações hospedeiro‐simbionte, potencialmente comprometendo a resistência dos hospedeiros à invasão de patógenos. Na Mata Atlântica Brasileira, um hotspot de biodiversidade, estudos prévios identificaram a seca como um fator chave que desestabiliza o microbioma cutâneo, contribuindo para a mortalidade de anfíbios devido ao fungo aquático patogênico *Batrachochytrium dendrobatidis* (Bd). No entanto, estudos observacionais frequentemente não conseguem separar o efeito direto da umidade sobre o crescimento do Bd e o aumento da atividade dos anfíbios durante as estações reprodutivas úmidas. Utilizando mesocosmos em campo, testamos o impacto das condições de seca no uso de microhabitat dos anfíbios hospedeiros, na dinâmica da doença causada pelo Bd e na composição e função antifúngicas das comunidades bacterianas cutâneas. Cada mesocosmo abrigou densidades ecologicamente realistas de *Brachycephalus pitanga,* o micro‐endêmico sapinho pingo‐de‐ouro. Submetemos metade dos mesocosmos a condições de seca utilizando coberturas de lona translúcida. Identificamos os indivíduos através de suas marcas únicas na pele e coletamos swabs cutâneos a cada duas semanas durante três meses. Em seguida, utilizamos técnicas moleculares para quantificar as cargas do Bd e caracterizar a diversidade e composição bacteriana da pele dos anfíbios ao longo do tempo. Nossos resultados indicam que, embora a seca reduza as cargas de infecção por Bd nos anfíbios, esse efeito é parcialmente contrabalançado pelo aumento do uso de áreas encharcadas nos mesocosmos e pela desestabilização do microbioma protetivo da pele dos anfíbios. Este estudo fornece uma nova perspectiva sobre os efeitos em cascata das mudanças climáticas no comportamento de vertebrados, nas interações hospedeiro‐simbionte e na dinâmica de doenças.

## 1 Introduction

A major goal of climate change research is to understand how disturbances, such as drought, may perturb complex natural systems across scales of biological organization (Davis et al. 2021; Vicente‐Serrano et al. 2020). Organisms can dampen the effects of rapid environmental disruption via behavioral plasticity (Beever et al. 2017), including altering microhabitat use (Scheffers et al. 2014). Such behavioral shifts can modify exposure and susceptibility to pathogens, further influencing disease dynamics as species respond to climate and land use change (Gallana et al. 2013; Harvell et al. 2002). For example, projections indicate that drought in the United States over the next 30 years could triple the number of West Nile virus cases in some regions by altering infection prevalence in birds through changes in avian reproduction, congregation patterns, and immunity (Paull et al. 2017). Shifts in environmental conditions can also alter host‐associated microbial communities, which are known to affect disease susceptibility in various marine and terrestrial systems (Egan and Gardiner 2016; Li et al. 2023). Despite this, few studies have experimentally tested how drought‐induced changes in wildlife behavior and microbial symbionts interact with pathogen transmission, leaving critical gaps in our understanding of these mechanisms.

Fungal diseases, which are particularly sensitive to environmental changes, have emerged as significant threats to global health (Williams et al. 2024). Factors such as increased international trade, altered precipitation patterns, rising global temperatures, and more frequent severe weather events are expected to change their strain evolution and spread (Singh et al. 2023). These diseases affect a wide range of organisms, including crops, wildlife populations, and even humans, posing risks to biodiversity (Fisher et al. 2020). Particularly devastating is *Batrachochytrium dendrobatidis* (Bd), a microscopic fungal pathogen that has significantly contributed to the imperilment of amphibians globally (Fisher and Garner 2020). Between 2004 and 2022, over half of all amphibian IUCN Red List category escalations were attributed to either climate change effects or disease (Luedtke et al. 2023).

**FIGURE 1:**
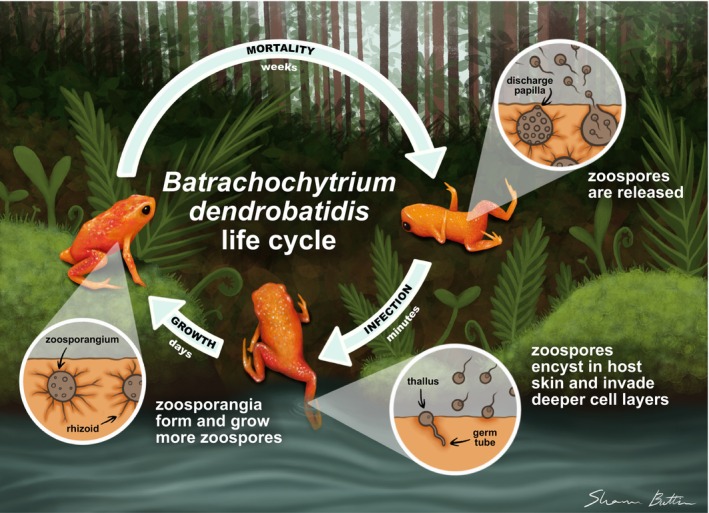
An illustration of the *Batrachochytrium dendrobatidis* (Bd) life cycle with our model host *Brachycephalus pitanga* . Bd begins its life cycle as a motile, waterborne zoospore that chemotactically moves toward host skin, encysts, and becomes a thallus. It then forms a germ tube to reach deeper layers of the skin and develops into a zoosporangium. Rhizoids grow to facilitate nutrient absorption and anchor the zoosporangium. As the zoosporangium matures, it produces new zoospores, which are released once the discharge papilla dissolves, enabling further infection or environmental dispersal.

Bd is a waterborne pathogen that is sensitive to desiccation (Johnson et al. 2003), and laboratory experiments show that higher substrate moisture increases Bd‐related mortality in salamanders (Raffel et al. 2015; Weinstein 2009). Similarly, frogs without access to dry areas in experimental tanks experienced higher reinfection rates due to moist conditions facilitating the heightened persistence and release of zoospores on their skin (Bustamante et al. 2010; Carey et al. 2006; Figure 1). Under dry conditions, Bd may be less likely to successfully colonize and persist on hosts, given the need for water films on the skin for zoospore movement (Raffel et al. 2015). Observational field studies have linked higher precipitation with increased Bd prevalence and/or infection loads in Chile (Alvarado‐Rybak et al. 2021), Jamaica (Holmes et al. 2014), the United States (Beyer et al. 2015; Terrell et al. 2014), and Brazil (Lambertini et al. 2021; Ruggeri et al. 2018). However, these studies cannot decouple the direct effect of moisture on Bd growth and colonization from the effects of behavioral responses to infection, nor increased amphibian activity and transmission during the wet breeding season (Ruggeri et al. 2018).

Despite Bd's optimal growth in wet conditions, numerous Bd‐attributed amphibian declines have been associated with droughts, including in Puerto Rico (Burrowes et al. 2004), Venezuela (Lampo et al. 2006), the United States (Adams et al. 2017; Kupferberg et al. 2021), and Brazil (Moura‐Campos et al. 2021). Many mechanisms may contribute to drought‐related declines. One possibility is that increased host stress leads to a weaker immune response and heightened susceptibility to Bd (Kohli et al. 2019; Rohr and Palmer 2013; Rollins‐Smith 2017). Another is related to behavioral changes that could concentrate amphibians in wet areas to offset hydric stress, increasing host‐to‐host transmission and/or the frequency of encountering aquatic Bd zoospores (Adams et al. 2017; Kupferberg et al. 2021). For instance, the installation of the Kihansi Dam in Tanzania reduced wet microhabitat availability for *Nectophrynoides asperginis* (the Kihansi spray toad), causing individuals to cluster together (Channing et al. 2006; Weldon et al. 2020). This aggregation, combined with the introduction of the Bd‐CAPE lineage, led to the eventual extinction of Kihansi spray toads in the wild (Sewell et al. 2024).

A third plausible mechanism for drought‐related Bd declines involves amphibian cutaneous bacterial communities, a key component of the skin microbiome that varies with seasonal precipitation changes (Varela et al. 2018). Bd infects the skin, and thus skin‐associated microbial communities are an important first line of defense against infection and can correlate with disease outcomes in amphibians (Hughey et al. 2022; Rebollar et al. 2016, 2020). Many members of the amphibian skin microbiome have been characterized and their Bd‐inhibitory function tested using microplate challenge assays (Woodhams et al. 2015), and there is evidence that these functions are relevant in field settings (Goodwin et al. 2022). In Brazil, a short‐term drought was linked to a Bd‐related die‐off of *Brachycephalus rotenbergae*, a species of terrestrial pumpkin toadlet (Moura‐Campos et al. 2021). Subsequent research revealed that the drought may have disturbed the toadlets' skin microbiomes and decreased the relative abundance of known Bd‐inhibitory bacteria on their skin, contributing to the die‐off (Buttimer et al. 2024). Few manipulative experiments have tested how drought modulates these mechanisms and alters host susceptibility and disease in natural settings.

**FIGURE 2:**
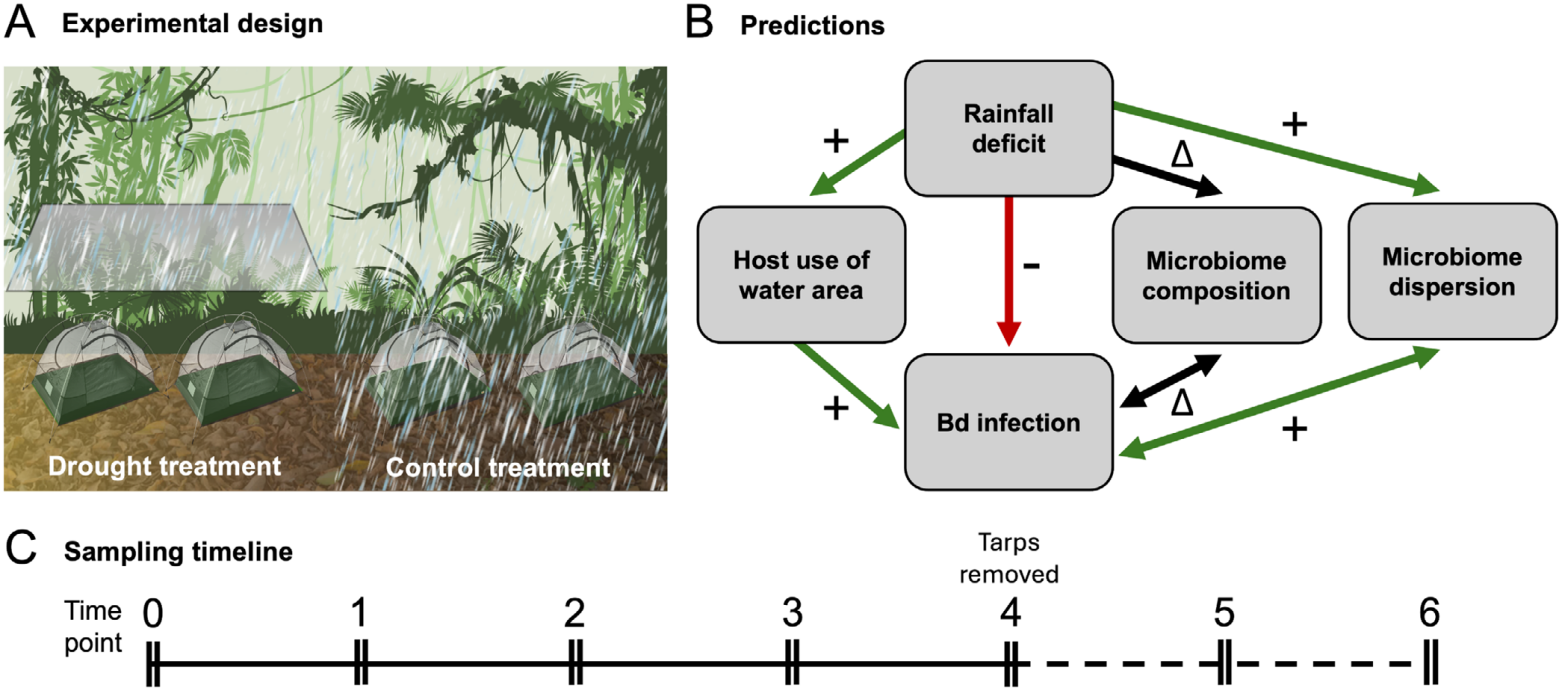
(A) Experimental design of one of five blocks of enclosures. (B) Flow chart showing the predicted relationships between variables. *Batrachochytrium dendrobatidis* (Bd) infection refers to Bd loads and/or presence on hosts. Hypothesis 1: Middle red arrow. Hypothesis 2: Left green arrows. Hypothesis 3: Right black and green arrows. (C) Sampling timeline showing when *Brachycephalus pitanga* were swabbed (over two consecutive days per time point) and when the tarps were removed from the drought treatment enclosures. Intervals between sampling were 2 weeks for a total experimental duration of 12 weeks.

Here, we conducted a field experiment to test how short‐term drought affects Bd dynamics, host microhabitat use, and the amphibian skin microbiome. We designed ecologically realistic enclosures with leaf litter, a water‐filled depression containing natural concentrations of locally collected Bd, and representative population densities of the pumpkin toadlet *Brachycephalus pitanga* , the sister species of *B. rotenbergae*, which recently experienced a Bd‐related die‐off. We simulated drought by excluding rainfall from half of the enclosures using translucent tarps, sampled the frogs' skin for bacteria and Bd, and recorded host movement throughout the 3‐month experiment (Figure 2A). Our hypotheses are threefold: (1) simulated drought has a direct negative effect on Bd proliferation on hosts, (2) simulated drought causes *B. pitanga* to increase their use of the wet area of the enclosures, leading to increased Bd transmission and/or exposure, and (3) skin microbiome composition shifts in response to drought, shows signs of disturbance (i.e., increased microbiome dispersion), and has lowered predicted Bd‐inhibitory function, increasing host susceptibility to infection (Figure 2B). Combined, our results highlight how rainfall variability may shape the balance between pathogen suppression and transmission in tropical amphibians.

## 2 Materials and Methods

### 2.1 Study System & Focal Amphibian Species

Brazil's Atlantic Forest is a biodiversity hotspot that harbors ~10% of all described amphibian species (Haddad et al. 2013). This region also exhibits high *Batrachochytrium dendrobatidis* (Bd) prevalence (Lambertini et al. 2021). Parque Estadual Serra do Mar—Núcleo Santa Virgínia is a state park in the Atlantic Forest in the state of São Paulo, Brazil (−23.341°, −45.149°; 930 m elevation) with a mean annual precipitation of 2180 mm. The area is predicted to exhibit increased rainfall variability and more consecutive dry days in the coming years due to deforestation and climate change (Marengo et al. 2020; Webb et al. 2005, 2006). Núcleo Santa Virgínia is home to *B. pitanga* (Anura: Brachycephalidae), a micro‐endemic species of pumpkin toadlet. This direct‐developing species lacks an aquatic larval stage and is prone to Bd infection via pathogen spillover from infected aquatic hosts (Becker et al. 2019). *B. pitanga* lives at high densities in the forest leaf litter and is easy to spot due to its bright red‐orange skin. These attributes make *B. pitanga* an ideal study organism for a field experiment that maximizes ecological realism.

### 2.2 Experimental Design

The experiment consisted of twenty 2.2 × 2.3 m mesh enclosures (four‐person tents; Clostnature, Yiwu City, China), set up in groups (blocks) of four within 500 m of each other under the canopy inside continuous forest (Figure 2A). We covered two enclosures in each block with translucent tarps hung approximately 2.5 m above the ground with 1.5 m of clearance on either side of the enclosure pair. One pair of covered enclosures was excluded from analysis due to disturbance during the experiment, resulting in data for 18 enclosures. To facilitate drainage, soil/leaf litter respiration, and movement of microarthropod prey into the tents, we modified the enclosures by cutting a 1.8 × 1.0 m rectangle out of the bottom of each and covering it with a piece of fine mesh secured to the remaining fabric with silicone. We did not observe water entering the tents from underneath. We cleared forest leaf litter before placing the enclosures on the ground, and we sifted through the leaf litter to ensure it was free of amphibians, then added the material back into each enclosure until the floor was completely covered.

We also added a water‐filled depression (0.20 × 1.80 × 0.08 m) using an impermeable tarp placed 0.20 m from one door and filled it with rainwater and leaf litter to allow frogs to climb in and out. To reflect naturally occurring Bd concentrations in the water, we used samples from an ongoing parallel community‐level field survey where we sampled *Dendropsophus minutus* , a Bd‐tolerant treefrog species commonly carrying Bd and shedding it in permanent ponds and temporary puddles throughout Núcleo Santa Virgínia (Becker and Zamudio 2011). At each time point, we added 10 mL of rainwater to sterile plastic bags (Whirl‐Paks; Nasco, Kellogg, ID) that held individual *D. minutus* for 8 h to pool low, naturally occurring concentrations of the local Bd genotype. We pooled the water from all bags and added 100 mL to the water‐filled depressions in 10 of the enclosures after each sampling period. This allowed us to maintain ecological realism in our experiment and avoid artificially inoculating frogs with Bd. We swabbed 10 random *D. minutus* at each time point to estimate Bd prevalence and load per frog (proportion infected = 0.867, mean Bd load = 130.543 ITS copies). All individual *D. minutus* were immediately released at the capture location. Importantly, we did not use stream or pond water to prevent uneven variation of environmental Bd concentration and environmental bacterial diversity over time and among treatments. We added additional rainwater to the water‐filled depressions as needed to maintain consistent water levels between treatments.

We caught 160 locally abundant *B. pitanga* for the experiment by gently raking through the leaf litter in and around the study location. We guided toadlets into sterile Whirl‐Paks without touching them and added some nearby leaf litter to reduce animal stress levels. We assigned each toadlet an ID number and took photographs for individual identification throughout the experiment, as they have unique skin patterns (Figure S1). We then rinsed the frogs with distilled water to remove transient bacteria (Culp et al. 2007) and swabbed each one with a sterile rayon swab (MWE‐113 Dryswab, Fine Tip; Medical Wire & Equipment, Corsham, United Kingdom). Swabbing consisted of five strokes on the dorsum, five strokes on each side of the venter, and five strokes on each arm and each leg. We stored swabs in sterile tubes containing 200 μL of DNAShield (Zymo Research, Irvine, CA, USA) and kept them frozen until DNA extraction. We swabbed all frogs before the experiment began on October 7, 2022, and no frogs had detectable Bd loads at the start of the experiment.

We randomly assigned eight individuals of *B. pitanga* to each enclosure, representing a representative density for this species. This species is not sexually dimorphic, and all animals were adults (over 10 mm snout‐vent length). Every 2 weeks, we recaptured, identified, and swabbed each individual *B. pitanga* . Due to logistical constraints, we added frogs to and sampled half of the enclosures on the first day and the remaining half the second day, repeating this biweekly for a total duration of 12 weeks (time points 0 through 6; Figure 2C). To assess host and host‐microbiome resilience to short‐term drought, we removed the tarps from the drought enclosures 8 weeks into the experiment (after sampling for time point 4; Figure 2C).

We collected spatial use data by visiting each enclosure daily and recording how many frogs could be seen in each of the 16 sections (Figure S2A). If no frogs were seen, we opened the enclosures and carefully moved leaf litter until we found at least two frogs per enclosure, then stopped to minimize disturbance. We removed dead frogs (*n* = 5) as soon as they were encountered. To account for differences in the total number of frogs in each enclosure (due to deceased or missing frogs) when analyzing count data, we calculated an adjusted spatial use metric. This metric was derived by multiplying the mean number of frogs in the water section over the 2 weeks preceding the time point of interest by the enclosure's maximum occupancy (eight) and dividing by the actual number of frogs present in the enclosure throughout the time point. The experiment concluded on January 3, 2023, after a total duration of 3 months.

Núcleo Santa Virgínia collects daily rainfall measurements, which we obtained from the park (Fundação Florestal 2023). We then used this data to calculate rainfall metrics for enclosures with and without tarps. While tarps covered the drought enclosures, we recorded the daily rainfall reaching them as 0 mm. For each sampling date, we calculated daily precipitation and 30‐day cumulative rainfall deficit, or the running difference in cumulative precipitation between the drought and control treatments over the past 30 days (Buttimer et al. 2024). The total cumulative rainfall difference between control and drought enclosures over the 2 months that the tarps were in place was 512 mm. Since 1970, the longest consecutive dry period recorded in the nearby city of Ubatuba (9 km from the study site) during this timeframe was 14 days, while the average duration of consecutive dry days was only 2.5 days (Centro Tecnológico de Hidráulica e Recursos Hídricos 2023). Leaf litter in the control enclosures remained moist throughout the study, whereas leaf litter in drought enclosures became and remained dry. This suggests that our simulated drought represented an extreme drying event for this region.

We installed a HOBO UA‐002‐64 Temp/Light Pendant (Onset, Bourne, MA, USA) between each pair of enclosures that recorded the ambient temperature three times per day. During the experiment, average temperatures in the control enclosures were 17.5°C (±2.3°C SD) and 17.7°C (±2.2°C SD) in the dry enclosures. Temperatures remained within permissive growth temperatures for Bd throughout the whole experiment (min.: 10.8°C, max.: 22.8°C; Piotrowski et al. 2004).

### 2.3 Sample Processing

Detailed descriptions of swab DNA extractions, Bd qPCR, and 16S rRNA microbiome library preparation can be found in the Supporting Information S1.

### 2.4 Microbiome Data Processing

We used Quantitative Insights into Microbial Ecology 2 (QIIME2) v2023.7 (Bolyen et al. 2019) to join forward and reverse reads (trimmed to 250 and 160 base pairs, respectively) and clustered reads from each run into Amplicon Sequence Variants (ASVs) using the Dada2 pipeline with default parameters (Callahan et al. 2016). Then, we merged the four sequencing libraries and exported a phylogenetic tree from QIIME2. We assigned taxonomy to the ASVs using the Greengenes2 V4 classifier (2022.10 backbone; McDonald et al. 2023) and removed chloroplasts, mitochondria, and sequences unassigned at the phylum level. To decontaminate sequences, we used the prevalence method of the R package *microDecon*, which uses proportions of contaminant ASVs in negative control samples to remove reads identified as contaminants (McKnight et al. 2019). To filter potential sequencing errors, we excluded ASVs comprising < 0.001% of the 14,361,495 total reads (Bokulich et al. 2013; Douglas et al. 2021).

Before rarefaction, we matched our ASVs to known bacteria whose metabolites have been tested for Bd‐inhibition via microplate challenge assays (Woodhams et al. 2015). We filtered our representative sequence file to the retained ASVs, then used Vsearch to match these sequences to 16S rRNA sequences of bacteria in the full AmphiBac database and to the strict inhibitory subset of the database (v.2023.2; Bletz et al. 2023). We then calculated the number of reads matching the inhibitory and full databases and created a closed‐reference metric for putative inhibition by dividing the number of reads classed as Bd‐inhibitory by the number of reads matching the full database containing all tested bacterial isolates. Due to substantial variation in representation in the database between samples, this approach should allow for a less biased comparison. Finally, we rarefied sequences at a depth of 4000 reads based on the rarefaction curves produced in QIIME 2 (Figure S3). We performed all other downstream analyses in R version 4.3.0 (R Development Core Team 2023).

### 2.5 Statistical Analysis

#### 2.5.1 Bd Loads and Presence

We ran two generalized linear mixed models (GLMMs) to investigate how Bd loads and presence were affected by the fixed effects of 30‐day cumulative rainfall deficit, the adjusted average number of hosts near water during the prior time point, and their one‐level interaction using the package *glmmTMB* (Brooks et al. 2022). Time point 0 was excluded from these models because behavioral data could not be collected prior to the introduction of frogs into the enclosures. Both models included a random effect of frog ID nested within enclosure block. We used a GLMM with a negative binomial distribution to model Bd loads. The Bd loads were rounded to the nearest integer using the ceiling() function to ensure that low loads were counted as positives, and the model equation is as follows: Bd load ~ (30‐day cumulative rainfall deficit) × (adjusted mean number of hosts near water) + (1|block/frog ID). We fitted a second GLMM to analyze the binary response variable, Bd presence, using a binomial distribution with a logit link function: Bd presence ~ (30‐day cumulative rainfall deficit) × (adjusted mean number of hosts near water) + (1|block/frog ID). We verified model assumptions using simulated residuals from the *DHARMa* package (Hartig and Lohse 2022).

To assess the general effect of rainfall exclusion tarps on Bd loads and presence, we ran an additional pair of GLMMs using *glmmTMB*. Both models used the same model families and random effect structures as above but included a single, binary fixed effect for whether the tarps were used during each time point.

#### 2.5.2 Skin Bacterial Communities

We identified core ASVs present in > 80% of samples using the R package *microbiome* (Lahti and Shetty 2023). To determine which ASVs best characterized each treatment at time point 3 (the time point during which there was the greatest difference in the proportion of Bd‐inhibitory reads between treatments), we ran LEfSe (Linear Discriminant Analysis [LDA] Effect Size) using the R package *microbiomeMarker* (Cao 2023). We used an LDA score cutoff of 3.4 and a Kruskal–Wallis test and Wilcoxon test cutoff of *p* < 0.01 in order to narrow down the number of ASVs. We calculated alpha diversity metrics (ASV richness and Shannon diversity) using the *vegan* package (Oksanen et al. 2020). We tested for pairwise differences in the proportion of Bd‐inhibitory reads between treatments at each time point using a Kruskal‐Wallis test from the *ggpubr* package (Kassambara 2023). We ran another two GLMMs for Bd load and Bd presence as above, but with the ratio of putative Bd‐inhibitory bacterial reads as the only fixed effect in each to assess whether this ratio was related to Bd infection.

We calculated beta diversity metrics (Bray–Curtis and Jaccard distances between microbiome samples) and Principal Coordinates Analysis (PCoA) axis values using the *phyloseq* package (McMurdie and Holmes 2013). To visualize the changes in average bacterial community composition through time, we calculated average PCoA axis values for each combination of time point × treatment and plotted the trajectories in *ggplot2* (Wickham et al. 2022). We ran a permutational multivariate analysis of variance test (PERMANOVA) on samples from time points 0 through 4 (i.e., before tarp removal) to test whether Bd loads, treatment, time point, and their interaction explained differences in bacterial community composition. The test was stratified by frog ID nested within enclosure block. We then used the betadisper function from the *phyloseq* package to calculate Bray–Curtis and Jaccard dispersion for each treatment × time point combination to quantify compositional variability between them, then tested for differences in dispersion between groups by running another PERMANOVA on the dispersion values.

We ran a second PERMANOVA to test whether microbiome composition before Bd infection but after the rainfall exclusion began (time points 1 and 2) varied with treatment, time point, clearance status [i.e., whether frogs (a) cleared their Bd infections or became undetectable during the experiment, (b) did not clear their infections—a Bd load > 0 at time point 6 or died, or (c) were never infected with Bd], and their interactions. Frogs with an unknown clearance status (*n* = 17) were excluded, and permutations were stratified by frog ID nested within enclosure block. To visualize differences in bacterial community composition between frogs of different clearance statuses from each treatment, we ordinated samples using a PCoA and plotted group ellipses using the *ggplot2* package.

#### 2.5.3 Relationships Between Short‐Term Drought, Host Behavior, Skin Bacteria, and Disease Dynamics

Finally, we employed piecewise structural equation modeling (PSEM) to check whether our predictions about the relationships between precipitation, host behavior, the skin microbiome, and Bd infection were supported (Figure 2B). In order to include both frog movement and microbiome data in the models, we only used data points retained after rarefaction from time points 1 through 6. As part of the PSEM, we fitted five GLMMs to investigate the relationships between 30‐day cumulative rainfall deficit (as a fixed effect) and each of the following response metrics: (1) microbiome composition (PCoA axis), (2) microbiome dispersion, (3) mean number of hosts near water, (4) rounded log_10_ Bd load, and (5) Bd presence. We ran each of the piecewise models using *glmmTMB* and visualized diagnostic plots using *DHARMa*. All models included a random effect term of frog ID nested within enclosure block.

The first two models in the PSEM modeled microbiome metrics (i.e., skin microbiome composition [Jaccard PCoA Axis 2] and microbiome dispersion [Bray–Curtis dispersion]) with 30‐day rainfall deficit and average number of frogs near water as fixed effects, using Gaussian distributions. The third model's response variable was the adjusted average number of frogs near water (rounded) as predicted by 30‐day rainfall deficit, for which we used a Poisson distribution. The fourth modeled rounded log_10_‐transformed Bd loads using a Poisson distribution, with 30‐day rainfall deficit and adjusted average number of frogs near water as fixed effects. Lastly, the fifth model used a binomial distribution with a logit link function to model the presence or absence of Bd and included 30‐day rainfall deficit and adjusted average number of frogs near water as fixed effects. After fitting each model, we constructed the PSEM using the package *piecewiseSEM* (Lefcheck et al. 2020). We included correlated errors between Bd load and microbiome composition, Bd load and microbiome dispersion, microbiome composition and microbiome dispersion, and Bd presence and Bd load. To compare the magnitude of direct and indirect (behavioral and microbiome) effects, we multiplied the standardized estimates along each path.

## 3 Results

### 3.1 Microhabitat Use and Bd Dynamics Respond to Altered Precipitation

**FIGURE 3:**
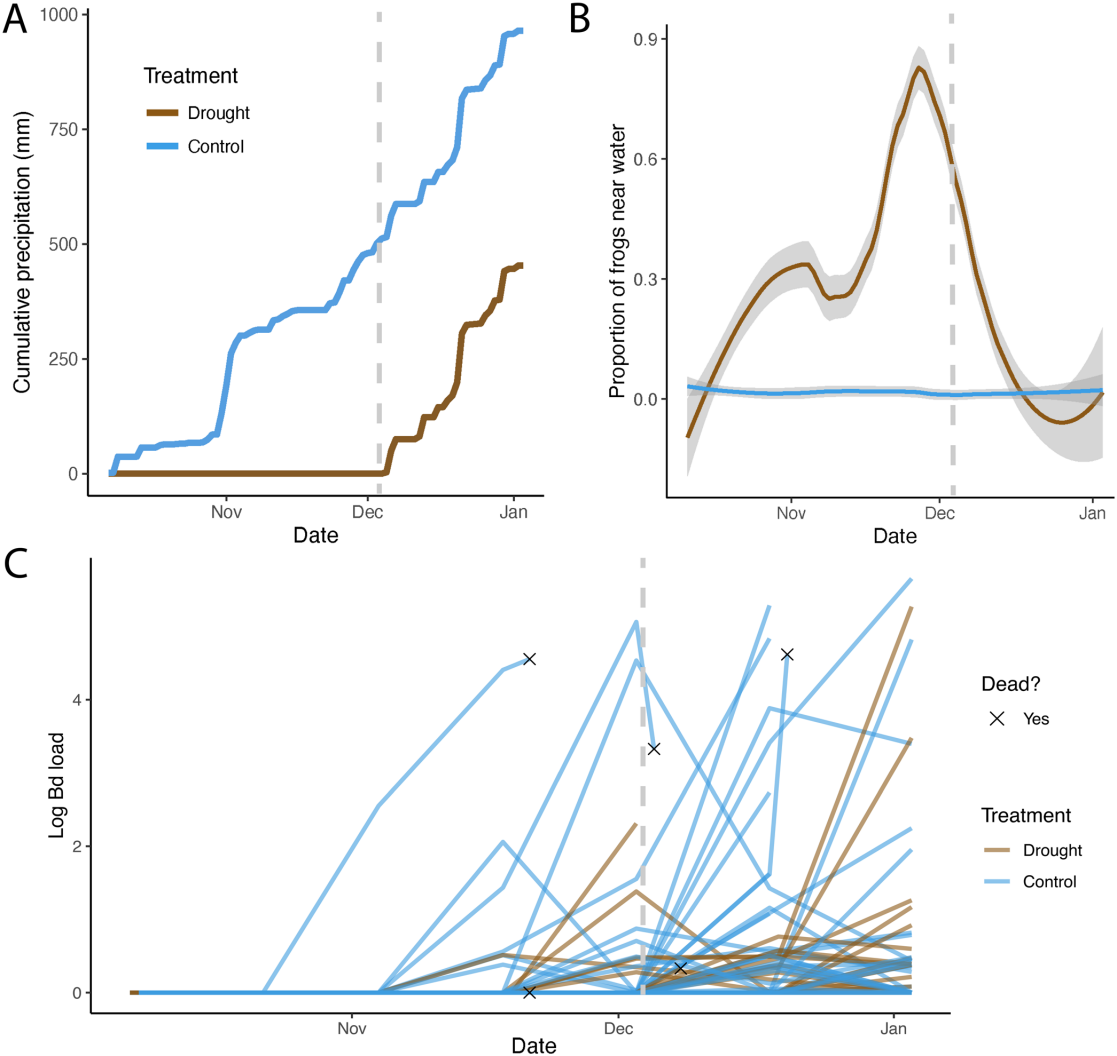
(A) Cumulative precipitation in control (blue) and drought (brown) enclosures. (B) The proportion of total *Brachycephalus pitanga* seen in and around the water‐filled depression in each enclosure. We applied a locally estimated scatterplot smoothing span of 0.6 to balance capturing the trend in *B. pitanga* activity while minimizing the risk of producing unrealistically negative values. (C) *Batrachochytrium dendrobatidis* (Bd) loads of individual toadlets over time. Vertical dashed lines indicate the date when the rainfall exclusion tarps were removed.

The microhabitat use of *B. pitanga* was related to the amount of precipitation reaching the enclosures (Figure 3A), with many more frogs choosing to spend time in and around the water‐filled depression in the drought treatment (Figure 3B). Following tarp removal from the drought treatments after time point 4, the proportion of frogs near water in the drought enclosures decreased, matching that in the control enclosures (Figure 3B). General patterns of space use among the 16 sections did not differ between the treatments except for in the section containing the water‐filled depression (Figure S3B–C). Overall, the presence of rainfall exclusion tarps decreased both *Batrachochytrium dendrobatidis* (Bd) loads and presence (Tables S1 and S2). More specifically, cumulative 30‐day rainfall deficit decreased both Bd loads (*β* = −0.029, *p* < 0.001) and Bd presence (*β* = −0.009, *p* < 0.001), while the average number of frogs found near water in the 2 weeks prior to sampling increased loads (*β* = 2.822, *p* < 0.001) and Bd presence (*β* = 0.936, *p* < 0.001; Table S3, Figure 3C). There was no detectable interactive effect between rainfall deficit and adjusted average number of hosts near water on Bd loads or presence (Table S3).

### 3.2 Core Skin Bacteria and Predicted Changes in Bd‐Inhibitory Function

**FIGURE 4:**
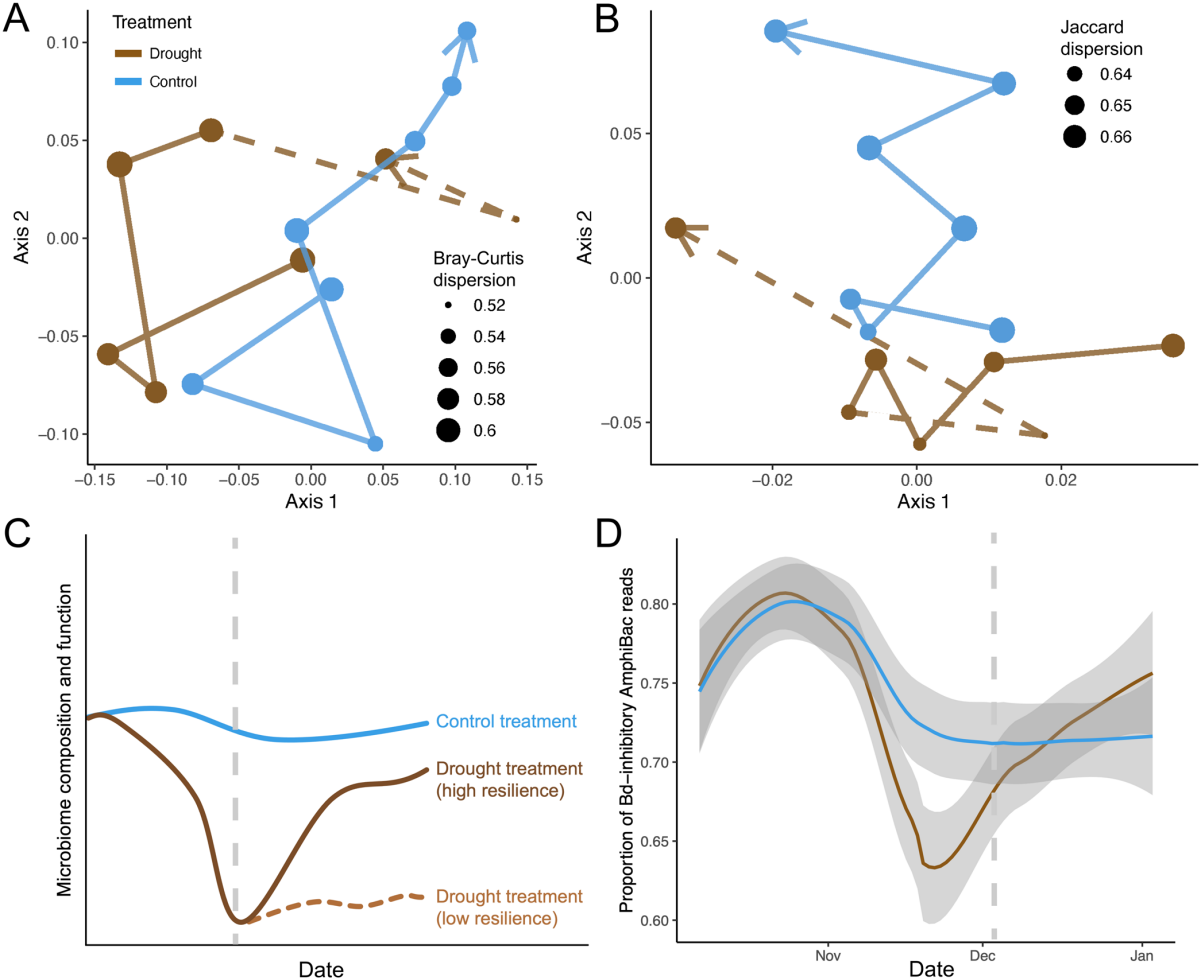
Trajectory plots showing the change in average skin bacterial community composition from time point 0 through 6 in each treatment using (A) PCoA of Bray–Curtis distances and (B) PCoA of Jaccard distances. Centroid size corresponds with average microbiome dispersion (variability), and dashed lines indicate time points after which tarps were removed from the drought enclosures. (C) Conceptual figure illustrating possible ways simulated drought could affect skin microbiome composition and/or *Batrachochytrium dendrobatidis* (Bd)‐inhibitory function. The blue line represents the average skin bacterial communities of *Brachycephalus pitanga* in enclosures under natural rainfall regimes, while the brown lines represent possible trajectories of skin microbial communities under simulated drought conditions and after tarps were removed (vertical dashed line). If the skin microbiome is resilient to disturbance, the community should return to a similar composition/function (brown solid line). If the community is not resilient, it may stay in an altered/less functional state (brown dashed line). (D) Results showing the proportion of reads attributed to Bd‐inhibitory bacteria over time in each treatment using the default locally estimated scatterplot smoothing span. The vertical dashed line indicates the date when the rainfall exclusion tarps were removed.

We detected three core bacteria in at least 80% of *B. pitanga* skin samples: a *Collimonas* sp. (unknown Bd‐inhibitory capacity), Burkholderiaceae sp. A592522 (presumed Bd‐inhibitory), and a *Bradyrhizobium* sp. (presumed non‐inhibitory). Bacterial richness and Shannon diversity did not vary much by treatment (Figure S4). Putative microbiome Bd‐inhibitory capacity changed throughout the experiment and was significantly lower in the drought treatment during time point 3 (Kruskal–Wallis *p* = 0.020) and returned to a level similar to the controls following tarp removal after time point 4 (Figure 4D). The drop in the proportion of Bd‐inhibitory reads in the drought treatment during time point 3 is driven by multiple Bd‐inhibitory bacteria (Figure S5). The proportion of Bd‐inhibitory reads was unrelated to both Bd presence (*β* = −0.902, *p* = 0.210) and rounded Bd loads (*β* = −2.463, *p* = 0.465), though both showed non‐significant negative correlations.

### 3.3 Effects of Treatment and Bd on Skin Microbiome Composition and Dispersion

Our PERMANOVAs indicate that treatment, time point, the interaction between treatment and time point, affect skin bacterial community composition, as measured by both Jaccard and Bray–Curtis distances (Table S4, Figure 4). PERMANOVAs on Jaccard dispersion values indicated significantly different dispersions between treatments (pseudo‐*F* = 9.912, *R* ^2^ = 0.146, *p* < 0.001), as was the case for Bray–Curtis dispersion (pseudo‐*F* = 8.815, *R* ^2^ = 0.132, *p* < 0.001). Figure 4A,B illustrate differences in average skin microbiome composition and dispersion over time. Differences in bacterial composition between treatments were most remarkable along axis 2 of Figure 4B, which we used in the PSEM.

In the drought treatment, skin microbiome composition during time points 1 and 2 (i.e., before widespread Bd infection in the tents) predicted the outcome of Bd infections later in the experiment (Table S5, Figure S6). This pattern was not repeated in samples from the control treatment, indicating that the composition of the skin bacterial community may only predict Bd infection outcomes under drier‐than‐average conditions (Figure S6).

### 3.4 Piecewise Structural Equation Modeling Reveals Direct and Indirect Effects of Drought on Bd Dynamics and the Skin Microbiome

**FIGURE 5:**
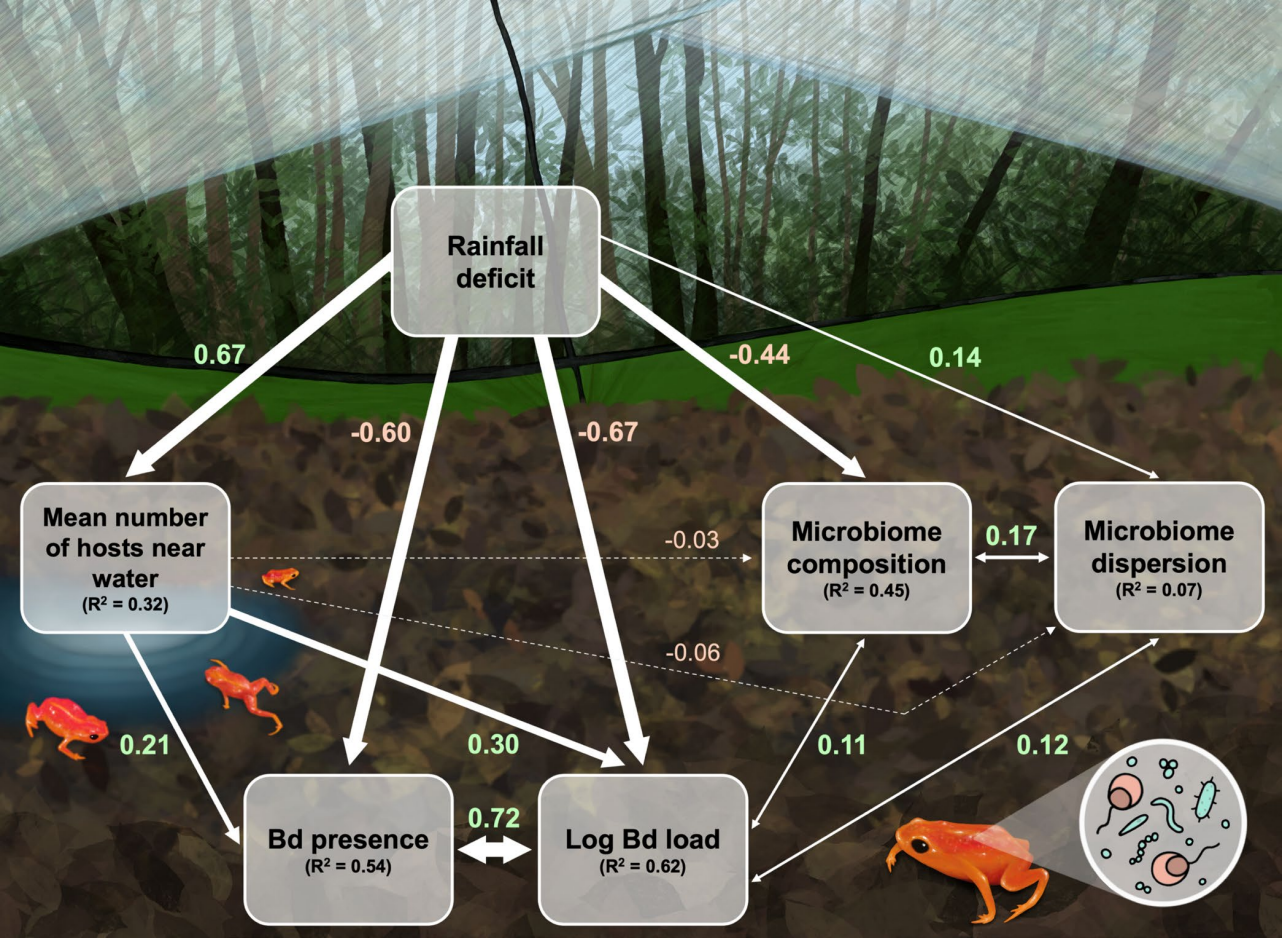
Piecewise structural equation model relating 30‐day cumulative rainfall deficit to the adjusted average number of *Brachycephalus pitanga* in and around the water‐filled depression during each time point, skin microbiome composition (Jaccard PCoA axis 2), microbiome dispersion (Bray–Curtis distances), log_10_‐transformed *Batrachochytrium dendrobatidis* (Bd) loads, and Bd presence. Double‐ended arrows represent correlated errors, while one‐ended arrows represent directed relationships among variables. Dashed arrows indicate non‐significant (*p* > 0.05) relationships. Arrow width is proportional to the standardized estimates, which are listed next to each arrow. Positive and negative standard estimates are shown in green and red, respectively. Conditional *R* ^2^ values are listed under each endogenous variable.

Results from the PSEM illustrate that simulated drought is a direct predictor of lower Bd loads and Bd presence (Global Fisher's *C* = 3.123, *p* = 0.538, df = 4, *n* = 634). Experimental drought also led to an increase in the number of hosts utilizing the water‐filled depression, which significantly predicted an increase in the number of frogs with Bd and their Bd infection loads (Figure 5). Microbiome composition along the Jaccard PCoA axis 2 significantly shifted with reduced rainfall and was correlated with Bd loads. Rainfall deficit also increased bacterial Bray–Curtis dispersion, which, in turn, correlated with higher Bd loads. Interestingly, only Bray–Curtis dispersion was affected by treatment, and it generally increased in the drought treatment, whereas Jaccard dispersion did not follow the expected trends (Figure S7). This indicates that drought increased the variability in ASV relative abundance more so than the presence or absence of ASVs. We found support for some of our microbiome hypotheses in response to drought, including a shift in bacterial community composition (Table S4, Figures 3 and 4), a signal of dysbiosis via Bray–Curtis, but not Jaccard dispersion (Figure 4, Figure S7), and lower predicted Bd‐inhibitory function (Figure 4D). Overall, the changes in host behavior and the shifts in the skin microbiome due to drought did not outweigh the direct negative effect of drying on Bd load, which was over three times stronger in total magnitude (Figure 5).

## 4 Discussion

We conducted a field experiment to test the impact of a short‐term drought on host behavior, the skin microbiome, and disease dynamics in a tropical vertebrate, showing that drought significantly alters host microhabitat use and may increase waterborne pathogen exposure and transmission. Results from our modeling framework indicated that experimental drought drove the pumpkin toadlet *B. pitanga* to preferentially utilize moist microhabitats, which serve as an environmental reservoir for the focal pathogen *Batrachochytrium dendrobatidis* (Bd), thereby increasing infection probability. Drought also altered the composition of the skin microbiome and led to a decline in the predicted Bd‐inhibitory function of these bacterial communities. Interestingly, despite these changes, toadlets in enclosures under the drought treatment generally exhibited lower Bd loads and prevalence, emphasizing the strong negative effect of drying on Bd proliferation. These findings demonstrate that drought events can influence waterborne disease dynamics in wildlife on multiple biological scales, highlighting the contrasting effects of drying on Bd, its host, and the skin microbiome, which collectively determine infection outcomes.

Experimental drought was linked to lower Bd infection loads in *B. pitanga* , which is consistent with what is known about the physiology and moisture requirements of Bd (Johnson et al. 2003). Bd is a waterborne fungus that requires moisture to reinfect host skin, so it is expected that moisture would increase Bd growth, resulting in lower survival of infected hosts under wet conditions in laboratory settings (Bustamante et al. 2010; Raffel et al. 2015). High‐elevation rainforests exhibit a high suitability for Bd, and frogs have experienced dramatic population declines related to chytridiomycosis in these habitats (Brem and Lips 2008; Catenazzi et al. 2014; James et al. 2015). As hypothesized, however, the negative effect of drying on Bd was partially offset by changes in host behavior under drought conditions that increased the number of hosts using the experimental pools to reduce hydric stress. Under more severe drought scenarios, this effect may be even stronger, as frogs may be forced to seek out water sources and remain there for longer time periods to avoid desiccation, leading to continuous exposure to Bd. These behavioral changes should increase the localized density of infected hosts as well as the frequency of environmental Bd transmission from the water to amphibians (Kupferberg et al. 2021). It is important to note, however, that we cannot determine whether the frogs in our experiment were being infected via environmental transmission (i.e., water, leaf litter), host–host contact, or a combination of these, and further studies would be needed to tease apart these potential pathways.

The drought‐induced behavior of *B. pitanga* , particularly their increased use of moist microhabitats, has significant implications for other amphibian species, especially terrestrial, direct‐developing species. These species lack an aquatic larval stage, have limited contact with water, and are often more susceptible to Bd—potentially due to a lack of acquired resistance from repeated pathogen exposure (McMahon et al. 2014; Mesquita et al. 2017). Their infection risk may be heightened as they seek out water sources and moist substrates during periods of rainfall deficit, unintentionally increasing their contact with infected aquatic species and environmental Bd reservoirs. Since indirect terrestrial transmission has been demonstrated experimentally (Burns et al. 2021), Bd in environmental substrates may drive the infection dynamics of direct‐developing species. Soil microbes can affect the invasibility of substrates by Bd (McGrath‐Blaser et al. 2024), and changes in climatic moisture and temperature may alter the Bd‐inhibiting potential of soil bacteria and environmental transmission of Bd.

Previous results indicated that drying events could potentially lead to shifts in the skin microbiome composition of the congeneric toadlet *Brachycephalus rotenbergae* (Buttimer et al. 2024). The present study supports this hypothesis, as average skin microbiome composition differed between treatment groups. These differences in composition were not strongly correlated with host infection intensity; however, skin microbiome composition during time points 1 and 2 correlated with infection outcomes under the drought treatment. Species with skin microbiomes that show might have greater Bd‐inhibitory function, like the direct‐developer *Haddadus binotatus* , may be more strongly affected by shifts in bacterial composition following disturbances (Martins et al. 2022). During the experimental drought, we detected a dip in the relative abundance of known Bd‐inhibitory taxa (identified through *in vitro* challenge assays), including the well‐known bacterium *Janthinobacterium lividum* , which is an example of a potentially functional member that has been tested as an amphibian probiotic with some success (Knapp et al. 2022; Kueneman et al. 2016). Encouragingly, the predicted Bd‐inhibitory capacity was resilient to drought as evidenced by a return to baseline levels after removing rainfall exclusion tarps, which coincided with a change in host microhabitat use to match the controls. The observed resilience of the skin microbiome to disturbance is promising, as host‐associated microbial communities may be able to adapt faster to climate change than their hosts (Kolodny and Schulenburg 2020; Petersen et al. 2023).

While our study provides key insights into climate‐disease interactions in amphibians, it is important to consider other unknown or unmeasured factors that may influence Bd dynamics and host‐microbiome interactions, such as changes in host physiology. For example, stress hormone levels can impact host immune and skin microbiome function (Gabor et al. 2015; Neely et al. 2023; Rollins‐Smith 2017), potentially altering the outcomes of Bd infection in amphibians during droughts. Infection with Bd can lead to increased ion loss and metabolic rates, particularly in smaller frogs, which, when combined with drought stress, could pose significant physiological challenges to hosts and result in higher mortality rates (Wu et al. 2018). Shifts in feeding behavior and the abundance of microarthropods may also play a role in host survival during droughts, although we did not visually observe any striking differences in body condition between treatments. Encouragingly, some individual toadlets from both treatments were able to clear low‐load infections, indicating that *B. pitanga* has the immune capacity and/or the skin microbiome function necessary to clear Bd infections when exposed to realistic numbers of zoospores under certain conditions.

Here, we provide additional evidence to suggest that the 2019 die‐off of *Brachycephalus rotenbergae* may have been triggered by drought‐induced changes in host behavior (i.e., microhabitat use) and increased susceptibility to Bd due to disruptions to the skin microbiome (Buttimer et al. 2024). The 2019 drought was followed by higher‐than‐average rainfall, which may have created a “perfect storm” for the proliferation of low‐level Bd infections on hosts. The Atlantic Forest, where these studies were conducted, is experiencing greater rainfall variability due to deforestation and climate change, resulting in more frequent droughts and floods (Marengo et al. 2020; Webb et al. 2006). These fluctuating conditions may exacerbate outbreaks by altering host behavior and subsequently promoting pathogen growth. This situation is reminiscent of outbreaks of African horse sickness (AHS) that are associated with the warm phase of the El Niño Southern Oscillation. During this phase, droughts cause animals to congregate near water sources, followed by heavy rainfall that enhances the reproduction of the AHS vector (Baylis et al. 1999). However, drought can also indirectly reduce exposure to pathogens, as observed in plains zebra ( *Equus quagga* ) populations where dry conditions decrease anthrax exposure through changes in host habitat choice (Huang et al. 2021). These examples highlight how climate variability can drive complex and context‐dependent effects on disease dynamics in wildlife, and our study is among the first to experimentally test these mechanisms in a wildlife‐pathogen system.

In conclusion, short‐term drought emerges as an important influence on host‐symbiont relationships, animal behavior, and disease dynamics. Our results show that drought suppressed Bd, but an increased host use of wet microhabitats and shifts in the protective skin microbiome partially offset the negative effect of drying on the pathogen. Population‐level disease outcomes will depend on the relative strength of these opposing mechanisms and will vary by host species and the degree of environmental disturbance. Conducting disease surveillance to detect and mitigate outbreaks, such as those observed in congener *B. rotenbergae* (Moura‐Campos et al. 2021), could help protect micro‐endemic species like *B. pitanga* during periods of unprecedented global change.

## Author Contributions

S.B., R.C.B., and C.G.B. designed the study. S.B., D.M., R.A.M., A.G.M.S., and C.G.B. coordinated and performed fieldwork, and S.B. conducted laboratory work and bioinformatics. S.B. analyzed the data and wrote the first draft of the manuscript with important contributions from C.G.B. All authors contributed to subsequent drafts.

## Disclosure

Any use of trade, firm, or product names is for descriptive purposes only and does not imply endorsement by the U.S. Government.

## Conflicts of Interest

The authors declare no conflicts of interest.

## Supporting information


Data S1.

## Data Availability

The data and code that support the findings of this study are openly available in Figshare at https://doi.org/10.6084/m9.figshare.29066894. Sequence data is publicly available in the NCBI Sequence Read Archive under accession PRJNA1170992.
