# Supplementary material for: Experimental Drought Suppresses Amphibian Pathogen Yet Intensifies Transmission and Disrupts Protective Skin Microbiome
Source: Glob Chang Biol. 2025 Jun 6;31(6):e70275. doi: 10.1111/gcb.70275 (PMC12142569; doi:10.1111/gcb.70275)
Supplement: Supplementary file 1 — Data S1. [file GCB-31-e70275-s001.docx]

**Supporting Information for**

**Experimental drought suppresses amphibian pathogen yet intensifies transmission and disrupts protective skin microbiome**

Shannon Buttimer*****^1,2^, Daniel Medina^1^, Renato A. Martins^3^, Ana Gabrielle Morais da Silva^4^, Wesley J. Neely^5^, Célio F. B. Haddad^3^, Graziella V. DiRenzo^6^, Alessandro Catenazzi^7^, Rayna C. Bell^8^, C. Guilherme Becker*****^1,2^

1. Department of Biology, and 2. One Health Microbiome Center, Center for Infectious Disease Dynamics, Huck Institutes of the Life Sciences, The Pennsylvania State University, University Park, PA 16802

3. Department of Biodiversity, Aquaculture Center (CAUNESP), and CBioClima, I.B., Universidade Estadual Paulista, Rio Claro, SP, Brazil

4. Centro Universitário Nossa Senhora do Patrocínio (CEUNSP), Itú, SP, Brazil

5. Department of Biology, Texas State University, San Marcos, TX 78666

6. U.S. Geological Survey, Massachusetts Cooperative Fish and Wildlife Research Unit, University of Massachusetts, Amherst, MA 01003

7. Department of Biological Sciences, Florida International University, Miami, FL 33199

8. Department of Herpetology, California Academy of Sciences, San Francisco, CA 94118

*****Shannon Buttimer, C. Guilherme Becker

**Email**: [shannon.buttimer@gmail.com](mailto:shannon.buttimer@gmail.com), [guibecker@psu.edu](mailto:guibecker@psu.edu)

*Any use of trade, firm, or product names is for descriptive purposes only and does not imply endorsement by the U.S. Government****.***

**DATA SHARING**

Sequence data is publicly available in the NCBI Sequence Read Archive under accession PRJNA1170992. All code and metadata files are available on figshare (doi.org/10.6084/m9.figshare.29066894).

**SUPPORTING METHODS**

**Sample Processing**

To extract DNA from swabs, we used GMax Mini Genomic DNA Kits (IBI Scientific, Dubuque, IA, USA) with a slight modification of the standard protocol. To aid in the lysis of Gram-positive cells, we added a lysozyme step to the protocol. The lysozyme buffer consisted of 3.15 g Tris-HCl, 0.744 g EDTA, and 12 mL Triton X-100 in 1L of distilled water, which we then autoclaved. We mixed 3.6 mg of lysozyme powder into 150 µL of buffer and sterilized it by passing the liquid through a 0.22 µM syringe filter before adding it to each sample. We then vortexed the samples and incubated them for 30 minutes at 37ºC. After adding the standard volume of Proteinase K, we incubated the swabs overnight at 60ºC to increase DNA yield (Caligiuri et al., 2019). To minimize biases based on sample collection date, we randomized the samples extracted each day and included an extraction control (sterile DI water) with each set.

We used a qPCR assay with Bd-specific primers (Boyle et al., 2004) and plasmid Bd standards (Pisces Molecular, Boulder, CO, USA) diluted from 2.6 x 10^6^ to 2.6 x 10^0^ Internal Transcribed Spacer (ITS) gene copies to quantify *Batrachochytrium dendrobatidis* (Bd) loads of skin swabs. We also included TaqMan Exogenous Internal Positive Control reagents to minimize false negatives by rerunning samples if IPCs did not amplify (Hyatt et al., 2007). Each 25 µL reaction included 12.5 µL TaqMan™ Fast Advanced Master Mix (ThermoFisher Scientific, Waltham, MA, USA), 1.13 µL of 20 µM ITS1-3 primer (ThermoFisher Scientific), 1.13 µL of 20 µM 5.8S Chytr primer (ThermoFisher Scientific), 1.20 µL of 5 µM Chytr MGB2 probe (ThermoFisher Scientific), 1 µL of 4x BSA (ThermoFisher Scientific), 0.83 µL of 10x Exo-IPC probe mix (ThermoFisher Scientific), 0.17 µL of Exo-IPC DNA (ThermoFisher Scientific), 2.05 µL of UltraPure water, and 5 µL of template DNA, Bd standard, or UltraPure water (negative control). We ran the reactions on a QuantStudio™ 3 (ThermoFisher Scientific) using the following program: 50ºC for 2 min, 95ºC for 10 min, then 50 cycles of 95ºC for 15 s and 62ºC for 1 min. We ran plates in duplicate and ran samples with mismatches in triplicate. We considered samples positive only if they tested positive on two plates. We averaged Bd loads between duplicate plates, then log_10_-transformed (average load + 1) to correct for non-normal residual distributions.

To identify bacterial Amplicon Sequence Variants (ASVs) present in each sample, we used PCR to amplify the V4 region of the 16S rRNA gene using a dual-index approach (Kozich et al., 2013). We amplified DNA in duplicate, using the following recipe for every sample: 12.2 µL of UltraPure water, 4 µL of 5X Phire reaction buffer (ThermoFisher Scientific), 0.4 µL of 2.5 mM dNTPs (ThermoFisher Scientific), 0.4 µL of Phire Hot Start II DNA Polymerase (ThermoFisher Scientific), 0.5 µL each of 10 µM barcoded forward and reverse primers (Integrated DNA Technologies, Coralville, IA, USA), and 2 µL of sample DNA. We ran duplicate PCR plates on Mastercycler^®^ Nexus Thermal Cyclers (Eppendorf, Hamburg, Germany) according to the following protocol: 98ºC for 3 mins, 38 cycles of 98ºC for 5 s, 50ºC for 5 s, and 72ºC for 15 s, then 72ºC for 3 min before holding at 12ºC. We included negative controls (UltraPure water) to check for contamination during PCR and randomly assigned samples to each library. We combined the duplicate PCR plates and visualized amplicons in 1% agarose gel to confirm amplification, then used SequalPrep Normalization Plates (ThermoFisher Scientific) to normalize amplicon concentrations. We purified the libraries using the QIAquick Gel Extraction Kit (Qiagen, Hilden, Germany). We sequenced the four 16S rRNA amplicon libraries using an Illumina MiSeq (Illumina, San Diego, CA, USA) at The Pennsylvania State University Huck Genomics Core Facility in University Park, PA, USA.

**SUPPORTING FIGURES**

**
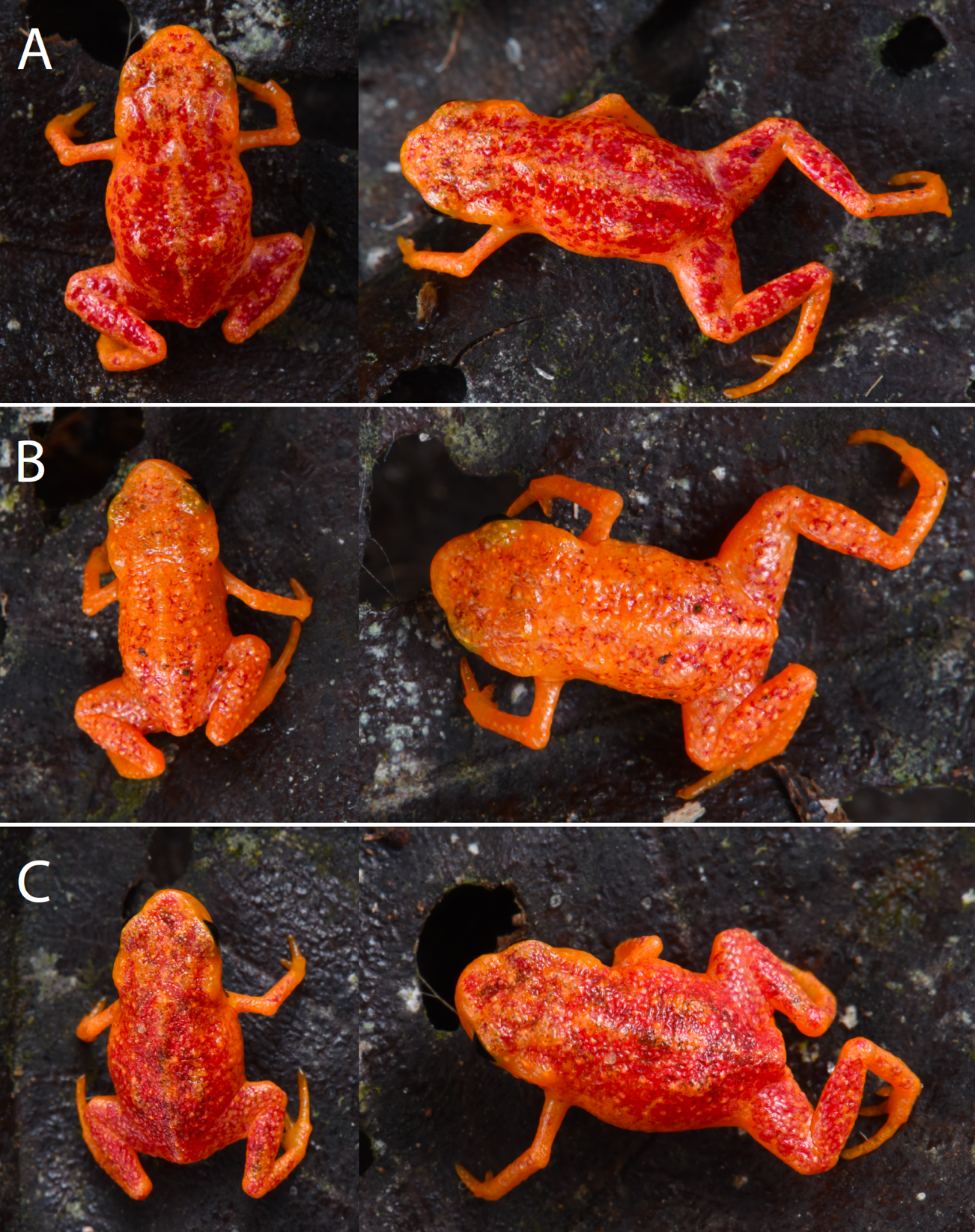
**

**Figure S1:** Example photographs of individual *Brachycephalus pitanga* (A, B, C). Unique skin patterning allowed us to identify to the individual level within each tent of eight toadlets.

**Figure S2: A)** Map of the enclosure floor with each of the 16 sections labeled. Green hatching represents the area where the enclosure floor had a mesh screen allowing for drainage, and blue represents the area of the water-filled depression. **B)** Spatial use of enclosures from time points zero through three, and **C)** spatial use of enclosures from time points four through six (after removing rainfall shelters from our drought treatment enclosures). We calculated normalized counts by dividing the count of *Brachycephalus pitanga* in each section by the total count of toadlets observed for each treatment and date.


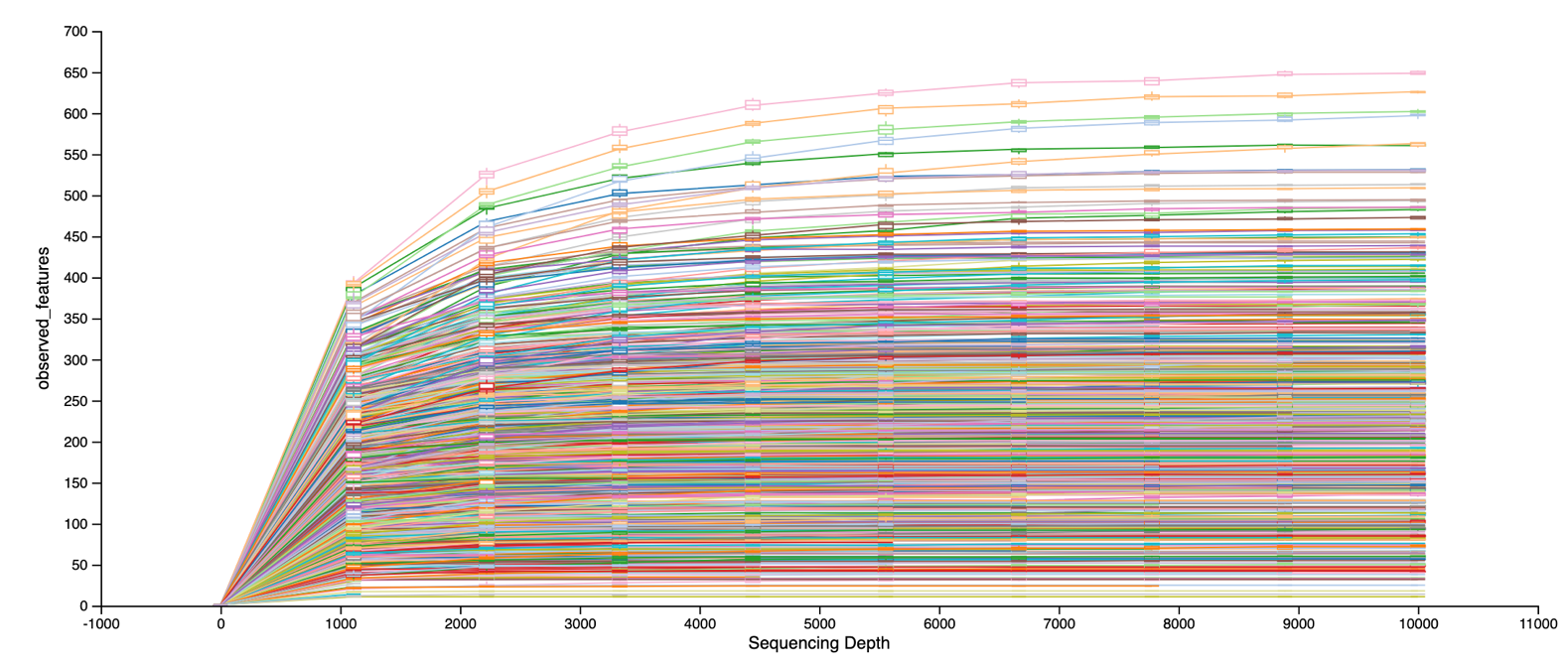


**Figure S3**: Rarefaction curves of all samples. We rarefied sequences at 4000 reads.

**
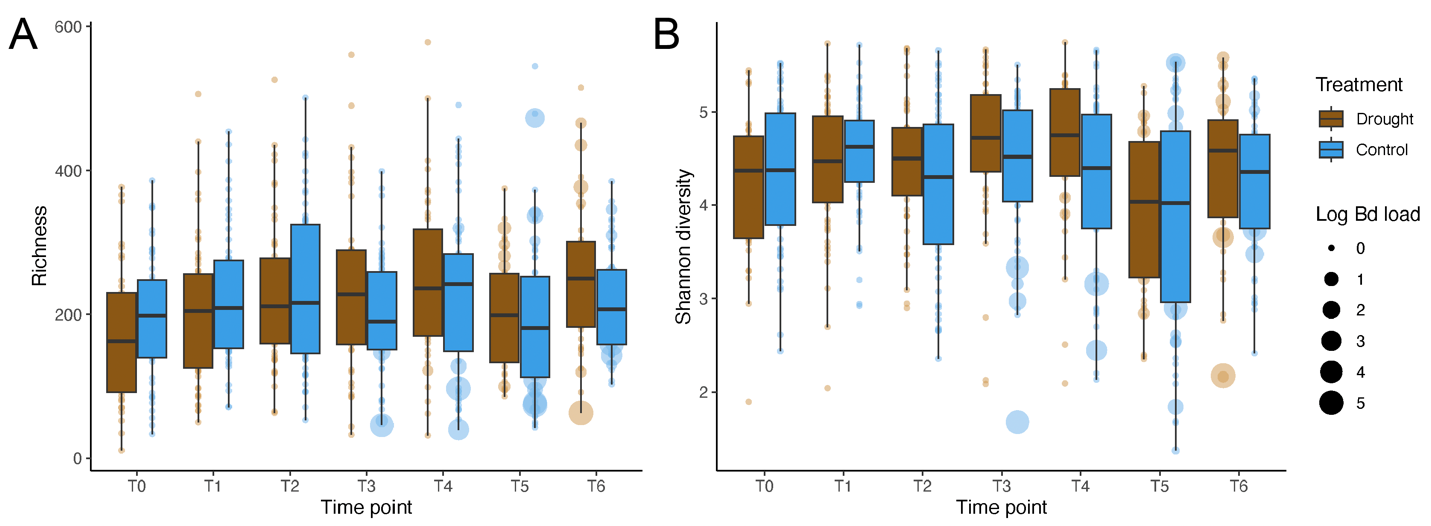
Figure S4**: **A)** Amplicon sequence variant (ASV) richness over time. There were no significant pairwise differences in richness between treatment groups. **B)** Shannon diversity of ASVs over time. Colors refer to treatment groups and points are scaled based on log_10_-transformed *Batrachochytrium dendrobatidis* (Bd) load.

**
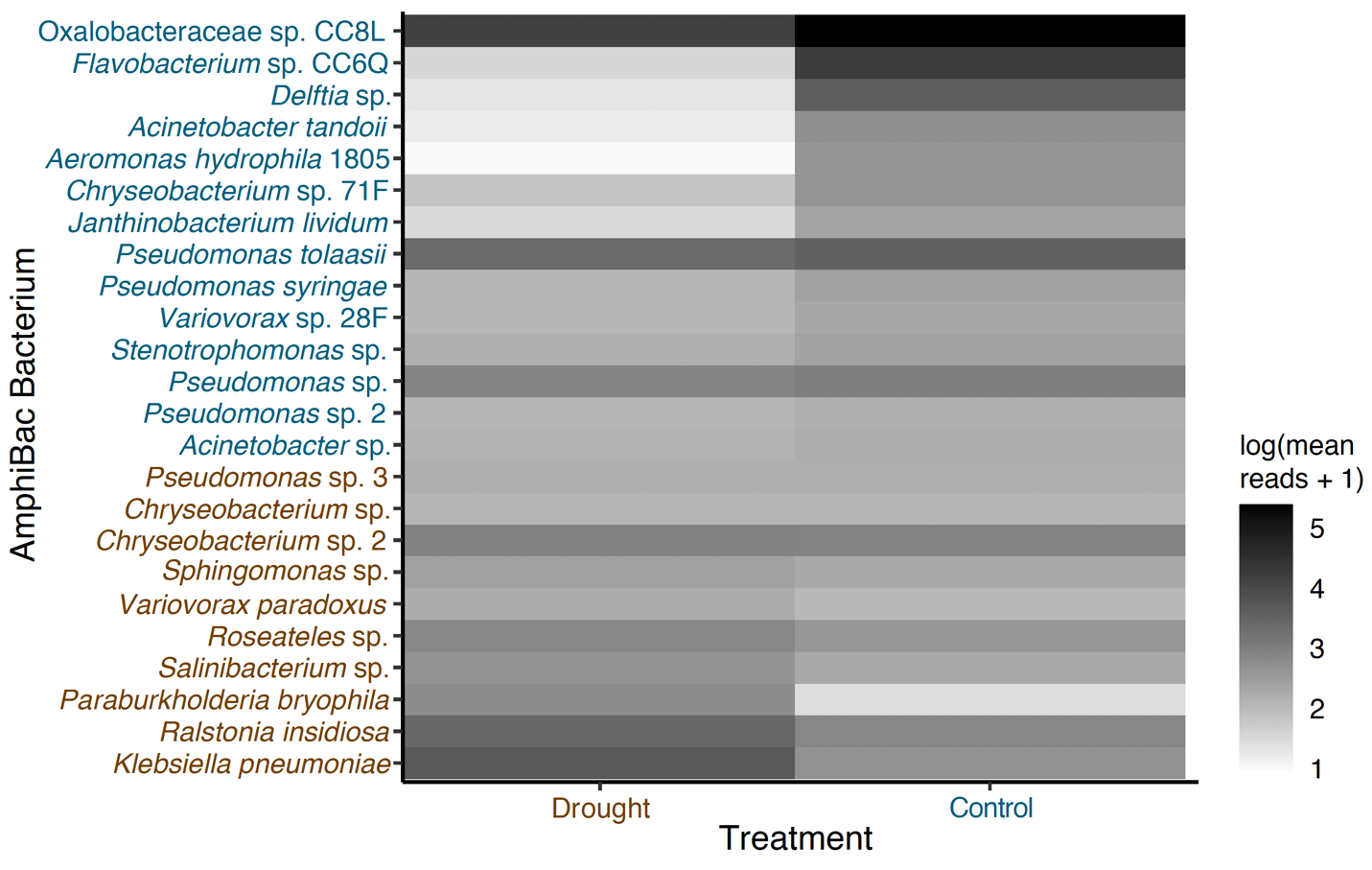
Figure S5:** Heatmap illustrating differences in relative abundance of predicted *Batrachochytrium dendrobatidis* (Bd)-inhibitory bacteria between treatment groups following during time point three. To narrow down to more dominant amplicon sequence variants (ASVs), only AmphiBac database bacteria with over 700 total reads are shown. Bacteria are ordered by the difference in mean reads between the control and drought treatments. Bacterium strain names are colored blue if they had a greater number of average reads in the control treatment and brown if they had a greater number of average reads in the drought treatment.

**
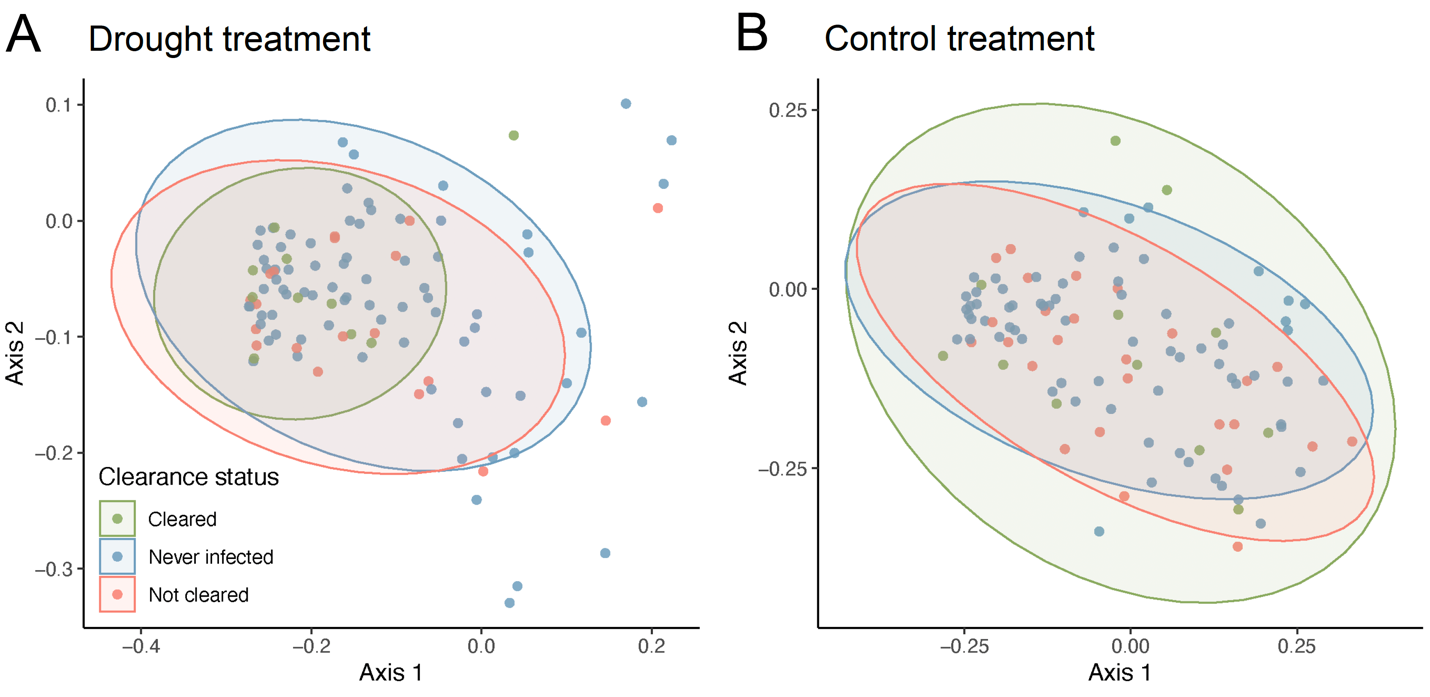
Figure S6**: Principal coordinates analysis of Bray-Curtis distances during time points one and two by *Brachycephalus pitanga* clearance status from **A)** Drought and **B)** Control treatments.

### **
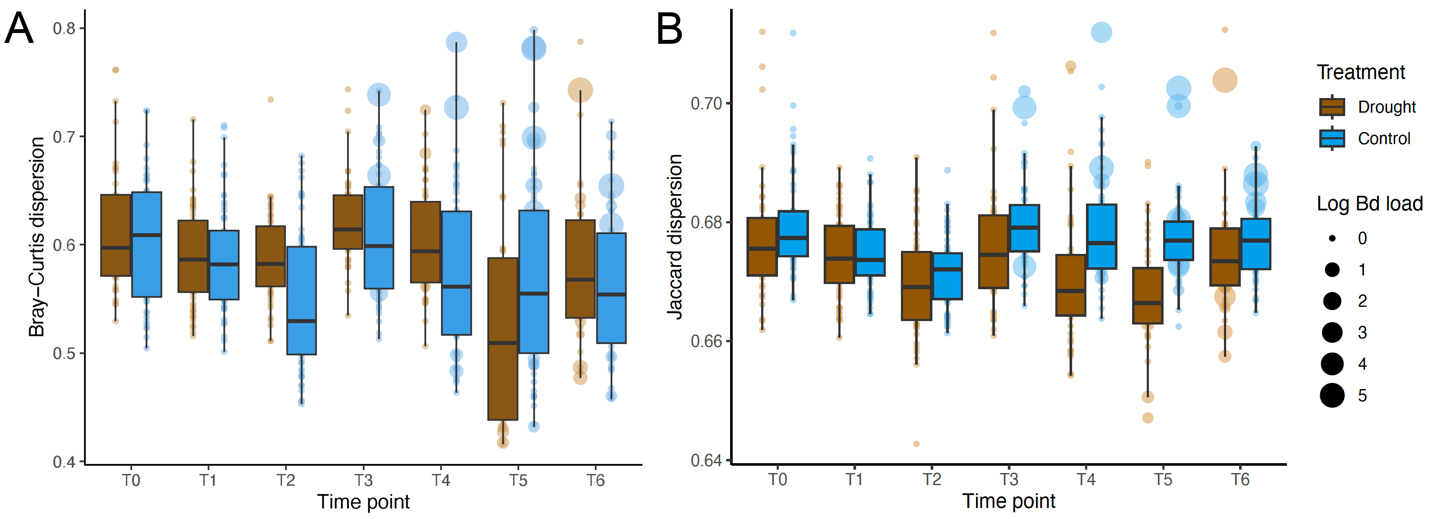
Figure S7: A)** Bray-Curtis dispersion over time, and **B)** Jaccard dispersion over time. Colors refer to treatment groups and points are scaled based on log_10_-transformed *Batrachochytrium dendrobatidis* (Bd) load.

**SUPPORTING TABLES**

**Table S1:** *Batrachochytrium dendrobatidis* (Bd) prevalence and loads for each time point and treatment. Loads are shown as log_10_-transformed number of internal transcribed spacer (ITS) copies + 1.

| **Time point** | **Treatment** | **Bd prevalence** | **Mean Bd load** |
| --- | --- | --- | --- |
| 0 | Control | 0.00 (0/89) | 0.00 |
|  | Drought | 0.00 (0/66) | 0.00 |
| 1 | Control | 0.00 (0/77) | 0.00 |
|  | Drought | 0.00 (0/64) | 0.00 |
| 2 | Control | 0.01 (1/74) | 0.76 |
|  | Drought | 0.00 (0/60) | 0.00 |
| 3 | Control | 0.08 (6/79) | 2.51 |
|  | Drought | 0.02 (1/57) | 0.02 |
| 4 | Control | 0.10 (8/77) | 3.29 |
|  | Drought | 0.10 (5/50) | 0.75 |
| 5 | Control | 0.31 (23/75) | 3.56 |
|  | Drought | 0.17 (8/47) | 0.14 |
| 6 | Control | 0.25 (17/68) | 3.88 |
|  | Drought | 0.24 (12/49) | 3.59 |

**Table S2:** Generalized linear mixed models assessing the effect of rainfall exclusion tarps on *Batrachochytrium dendrobatidis* (Bd) load (negative binomial distribution) and Bd presence (binomial distribution with logit link) from time points one through six (n = 777). Samples from dead *Brachycephalus pitanga* were excluded.

| **Response variable** | **Explanatory variable** | **Estimate** | **z-value** | ***p*-value** |
| --- | --- | --- | --- | --- |
| Bd load | Tarp presence | -6.910 | -5.945 | **<0.001** |
| Bd presence | Tarp presence | -2.198 | -4.635 | **<0.001** |

**Table S3:** Full-factorial generalized linear mixed models relating 30-day cumulative rainfall deficit, the mean number of *Brachycephalus pitanga* near water, and their interaction on *Batrachochytrium dendrobatidis* (Bd) load (negative binomial distribution) and Bd presence (binomial distribution with logit link) from time points one through six (n = 777). Samples from dead *B. pitanga* were excluded.

| **Response variable** | **Explanatory variable** | **Estimate** | **z-value** | ***p*-value** |
| --- | --- | --- | --- | --- |
| Bd load | Cumulative 30-day rainfall deficit (mm) | -0.029 | -6.575 | **<0.001** |
|  | Adjusted mean number of frogs near water | 2.822 | 3.425 | **<0.001** |
|  | Interaction | 0.016 | 1.268 | 0.205 |
| Bd presence | Cumulative 30-day rainfall deficit (mm) | -0.009 | -6.262 | **<0.001** |
|  | Adjusted mean number of frogs near water | 0.936 | 3.752 | **<0.001** |
|  | Interaction | 0.002 | 0.510 | 0.610 |

**Table S4**: Permutational multivariate analysis of variance (PERMANOVA) of Jaccard and Bray-Curtis distances, comparing composition by treatment, time point, and *Batrachochytrium dendrobatidis* (Bd) load. Samples from time points zero through four (n = 532) were permuted by *Brachycephalus pitanga* ID nested within enclosure block.

| **Distance metric** | **Explanatory variable** | **Pseudo-F** | **R^2^** | **P-value** |
| --- | --- | --- | --- | --- |
| Jaccard | Treatment  Time point  Treatment x time point  Bd load | 1.688  1.777  1.142  1.078 | 0.003  0.013  0.009  0.002 | **<0.001**  **<0.001**  **<0.001**  0.114 |
| Bray-Curtis | Treatment  Time point  Treatment x time point  Bd load | 7.329  6.086  1.748  1.303 | 0.013  0.043  0.012  0.002 | **<0.001**  **<0.001**  **<0.001**  0.075 |

**Table S5:** Permutational multivariate analysis of variance (PERMANOVA) of Jaccard and Bray-Curtis distances, comparing composition between different clearance statuses (cleared, not cleared, and never infected) from samples during time points one and two (n = 213). Permutations were stratified by *Brachycephalus pitanga* ID nested within enclosure block.

| **Distance metric** | **Explanatory variable** | **Pseudo-F** | **R^2^** | **P-value** |
| --- | --- | --- | --- | --- |
| Jaccard | Clearance status  Treatment  Time point  Clearance status x treatment  Treatment x time point  Clearance status x time point | 1.016  1.448  1.563  1.031  1.079  0.994 | 0.010  0.007  0.007  0.010  0.005  0.009 | **<0.001**  **<0.001**  **<0.001**  **<0.001**  **0.005**  0.580 |
| Bray-Curtis | Clearance status  Treatment  Time point  Clearance status x treatment  Treatment x time point  Clearance status x time point | 1.001  5.093  6.411  1.178  1.632  1.026 | 0.009  0.023  0.029  0.011  0.007  0.009 | **<0.001**  **<0.001**  **<0.001**  **<0.001**  **0.019**  0.196 |

**SUPPORTING REFERENCES**

Boyle, D. G., Boyle, D. B., Olsen, V., Morgan, J. a. T., & Hyatt, A. D. (2004). Rapid quantitative detection of chytridiomycosis (*Batrachochytrium dendrobatidis*) in amphibian samples using real-time Taqman PCR assay. *Diseases of Aquatic Organisms*, *60*(2), 141–148. https://doi.org/10.3354/dao060141

Caligiuri, L. G., Sandoval, A. E., Miranda, J. C., Pessoa, F. A., Santini, M. S., Salomón, O. D., Secundino, N. F. C., & McCarthy, C. B. (2019). Optimization of DNA extraction from individual sand flies for PCR amplification. *Methods and Protocols*, *2*(2), 36. https://doi.org/10.3390/mps2020036

Hyatt, A. D., Boyle, D. G., Olsen, V., Boyle, D. B., Berger, L., Obendorf, D., Dalton, A., Kriger, K., Hero, M., Hines, H., Phillott, R., Campbell, R., Marantelli, G., Gleason, F., & Colling, A. (2007). Diagnostic assays and sampling protocols for the detection of *Batrachochytrium dendrobatidis*. *Diseases of Aquatic Organisms*, *73*(3), 175–192. https://doi.org/10.3354/dao073175

Kozich, J. J., Westcott, S. L., Baxter, N. T., Highlander, S. K., & Schloss, P. D. (2013). Development of a dual-index sequencing strategy and curation pipeline for analyzing amplicon sequence data on the MiSeq Illumina sequencing platform. *Applied and Environmental Microbiology*, *79*(17), 5112–5120. https://doi.org/10.1128/AEM.01043-13
